# Enthusiasm for homework and improvement of psychological distress in subthreshold depression during behavior therapy: secondary analysis of data from a randomized controlled trial

**DOI:** 10.1186/s12888-015-0687-3

**Published:** 2015-11-25

**Authors:** Y. Hayasaka, T. A. Furukawa, T. Sozu, H. Imai, N. Kawakami, M. Horikoshi, On behalf of the GENKI Project

**Affiliations:** 1 Department of Health Promotion and Human Behavior, Kyoto University Graduate School of Medicine / School of Public Health, Yoshida Konoe-cho, Sakyo-ku, Kyoto 606-8501 Japan; 2 Department of Management Science, Faculty of Engineering, Tokyo University of Science, 1-3 Kagurazaka, Shinjyuku-ku, Tokyo 162-8601 Japan; 3 Department of Mental Health, University of Tokyo Graduate School of Medicine, Hongo, Bunkyo-ku, Tokyo 113-0033 Japan; 4 National Center of Neurology and Psychiatry, Kodaira, Tokyo 187-8551 Japan

**Keywords:** Enthusiasm, Cognitive behavioral therapy, Telephone cognitive behavioral therapy, Homework, Depression, Subthreshold depression, Structural equation modeling

## Abstract

**Background:**

Cognitive behavioral therapy (CBT) usually involves homework, the completion of which is a known predictor of a positive outcome. The aim of the present study was to examine the session-by-session relationships between enthusiasm to complete the homework and the improvement of psychological distress in depressed people through the course of therapy.

**Methods:**

Working people with subthreshold depression were recruited to participate in the telephone CBT (tCBT) program with demonstrated effectiveness. Their enthusiasm for homework was enhanced with motivational interviewing techniques and was measured by asking two questions: “How strongly do you feel you want to do this homework?” and “How confident do you feel you can actually accomplish this homework?” at the end of each session. The outcome was the K6 score, which was administered at the start of each session. The K6 is an index of psychological distress including depression and anxiety. We used structural equation modeling (SEM) to elucidate the relationships between enthusiasm and the K6 scores from session to session.

**Results:**

The best fitting model suggested that, throughout the course of behavior therapy (BT), enthusiasm to complete the homework was negatively correlated with the K6 scores for the subsequent session, while the K6 score measured at the beginning of the session did not influence the enthusiasm to complete the homeworks assigned for that session.

**Conclusions:**

Empirical data now support the practitioners of BT when they try to enhance their patient’s enthusiasm for homework regardless of the participant’s distress, which then would lead to a reduction in distress in the subsequent week.

**Trial Registration Number:**

ClinicalTrials.gov NCT00885014. April 20, 2009.

## Background

Subthreshold depression, sometimes also called subsyndromal depression or minor depression, refers to a depressive state that does not meet the full diagnostic criteria for major depressive disorder (MDD) [1]. It would be classified as Depressive Disorder Not Otherwise Specified in DSM-IV [2] or Other Specified Depressive Disorder in DSM-5 [3]. Patients with subthreshold depression often present with both depression and anxiety symptoms, albeit both under any diagnostic thresholds [4]. It has been increasingly recognized that subthreshold depression is highly prevalent in the general population [5] and in the primary care [6], is clinically relevant because it significantly affects the quality of life and functioning of the sufferers [7] and carries a high risk of developing major depressive disorder [8], and is societally important because it is associated with high economic costs [9].

Cognitive behavioral therapy (CBT), the psychotherapy for depression and anxiety with the largest number of randomized controlled trials (RCTs) supporting its efficacy [10, 11], has been shown to be effective for subthreshold depression [12] as well. In CBT, patients self-monitor their own behaviors, emotions and thoughts and also practice newly learned cognitive or behavioral coping skills outside the therapy sessions as homework. Homework is usually assigned in every session of CBT, asking patients to practice newly learned cognitive or behavioral skills and to generalise such skills to their daily situations in which their problems occur [13]. In general, homework has been shown to facilitate improvement in depression, anxiety or other client problems through CBT [14] but patients often find it difficult to complete the homework [15–19].

In order to understand and possibly facilitate the process of CBT, it is therefore important to examine which aspects of the homework are important in this therapeutic process. Client characteristics, therapist characteristics, characteristics of the task, and interrelationships among these components have been considered to influence the homework compliance [20]. Among the first components, the clients’ motivational level may be one of the important elements for enhancing homework compliance. Some psychological interventions, such as motivational interviews, are aimed at enhancing enthusiasm [21]. However, no study has yet examined whether the enthusiasm to complete homework assignments thus enhanced can lead to improved process and outcome of CBT.

Moreover, the extant studies of homework in CBT suffers one crucial methodological weakness. In the literature, homework compliance has usually been assessed either post-hoc after the treatment is over, thus risking the recall bias [22, 23], or only once out of the 10 or more sessions of the program, thus possibly not reflecting the overall compliance [24, 25]. Even when homework compliance was measured several times, only the average of those several values was used to predict the outcome of the treatment [26, 27]. To the best of our knowledge, no study to date has examined the session-by-session relationships between homework and an improvement through CBT.

We have previously conducted a randomized controlled trial of CBT administered via telephone among employees with subthreshold depression in a large company in Japan. This telephone CBT (tCBT) program was shown to have a large effect, in comparison with a waiting list control group, with an effect size of around 0.7 for the primary outcome of general psychological distress including depression and anxiety [28]. In this trial, we applied motivational interviewing techniques and measured the enthusiasm of the participants for completing homework at the end of each session.

The current study therefore focused on enthusiasm rather than compliance and aimed to examine the session-by-session relationships between the participants’ enthusiasm to complete the homework and the therapeutic outcomes using structural equation modeling (SEM). We hypothesized that high enthusiasm to complete the homework assignments would be associated with improvement of psychological distress in the subsequent session.

## Methods

**Fig. 1:**
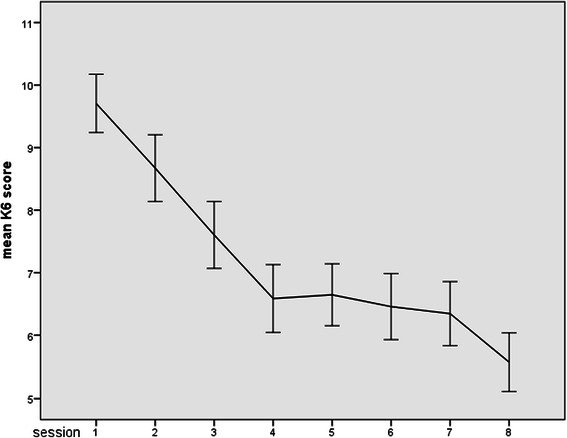
Session-by-session changes of mean K6 scores. The error bars represent the standard error

This study is a secondary analysis of data from a previous RCT. The original RCT was approved by the Ethics Review Committee of Nagoya City University Graduate School of Medicine, and the present re-analysis has been approved by the Ethics Committee of Kyoto University Graduate School of Medicine. The original report of this RCT adhered to CONSORT guidelines and the research design and methods of the RCT have been described in detail in a previous publication [28]. Summarized briefly, the study involved an RCT to compare the effectiveness of tCBT in addition to an Employee Assistance Program (EAP) and EAP alone for the treatment of subthreshold depression among workers at a large manufacturing company in Japan. At the initial screening, potential participants were asked to provide their written informed consent to fill in the screening questionnaire after full explanation of the purpose and procedure of the study. Then those who fulfilled the eligibility criteria were asked to provide their final informed consent to participate in the intervention study. Two thousand three hundred twenty-two employees were assessed for eligibility, of whom 145 met the eligibility criteria and invited to participate in the RCT. Twenty-seven declined and 118 finally took part in the original RCT. Fifty-eight were allocated to the tCBT arm and started the telephone sessions immediately. Sixty were allocated to the EAP alone arm (waiting list) and had to wait for four months, complete the end-of-treatment questionnaire and, if they still desired, began receiving the tCBT sessions (See Appendix: Figure 1)

### Participants

We defined subthreshold depression as depressive state scoring above the predefined thresholds on screening questionnaires but failing to reach the diagnostic threshold for major depression.

The inclusion criteria were as follows:

1. An age of 20–57 years at the time of study entry, (the retirement age was 60 and we took a period for follow-up into consideration)
2. Currently employed full-time (either regular or temporary),
3. Expected to be employed full-time for 6 months after the screening,
4. K6 scores greater than or equal to 9 at screening and greater than or equal to 5 at the first tCBT session (baseline),
5. Beck Depression Inventory-II (BDI2) [29] score of greater than or equal to 10 at screening, and
6. Participation in one or more sessions of the tCBT program.

The exclusion criteria were as follows:

1. Major depressive episode in the past month, as ascertained using the Composite International Diagnostic Instrument (CIDI) [30] (We did not exclude dysthymia or major depression in partial remission.),
2. Lifetime history of bipolar disorder, as ascertained using the CIDI,
3. Any substance dependence during the past 12 months, as ascertained using the CIDI,
4. Any other current mental disorder if it constituted the predominant aspect of the clinical presentation and required treatment not offered in the study,
5. Current treatment for a mental health problem from a mental health professional,
6. Sick leave for 6 or more days for a physical or mental condition in the past month, and
7. Expected to be on pregnancy leave, maternity leave, or nursing leave within 6 months after screening.

### Measures

#### Enthusiasm

The participants’ enthusiasm to complete therapy-related homework was estimated from the responses to two questions. The therapist asked the participants at the end of each session after setting the homework for the week, “How strongly do you feel you want to do this homework? (Q1)” and “How confident do you feel you can actually accomplish this homework this week?” (Q2). The participants responded using a scale of 0 to 10, with 0 representing no enthusiasm and 10 representing maximum enthusiasm. When there were more than one homework assignment from the session, the two questions were repeated for each homework assignment and the average score was used to represent the participants’ enthusiasm at that session.

In asking these questions, some motivational interviewing techniques were used, so that difficult homework assignments were broken down to smaller pieces and/or tips to troubleshoot potential barriers to completing homework were discussed between the therapist and the participant using Socratic questions (e.g., When the patient rating was high, for example, at 8, a Socratic question such as “I can see that you are quite willing/confident to do this homework. But what is in the remaining 2 points?” might further enhance the motivation of the participant and/or would call for collaborative troubleshooting for possible barriers to homework completion. When the rating was moderate, for example, at 5, a different question such as “What can we do to improve your rating by one or two points?” can be very helpful for both the participant and the therapist to collaboratively modify and clarify the homework task. And when the rating was very low, for example 2 or even 1, then a complete breaking down of the homework task would be called for.). The final scores were collected after the participants’ enthusiasm for the week’s homework would be enhanced as much as possible through motivational interviewing.

Cronbach’s alpha coefficient for the enthusiasm measure was 0.75 (95 % CI: 0.68 to 0.80). In general alpha coefficients of 0.7 or greater are considered to be represent satisfactory internal consistency reliability [31].

#### Psychological distress

The outcome was the K6 score, measured at the beginning of each session regarding the participant’s status for the preceding week. The K6 is a 6-item, self-reported index of psychological distress, including four questions about depression (hopelessness, depressed mood, anergia, and worthlessness) and two questions about anxiety (nervousness and restlessness) [32]. Each item is rated between 0 = none of the time and 4 = all of the time. The total score therefore ranged between 0 and 24. The Japanese version has been validated [33]. We administered the K6 as an initial screening instrument and used a cutoff of 8/9 according to the Japanese calibration study [33]. We also used K6 as a process measure throughout the course of tCBT. K6 usually measures psychological distress for the past 30 days but we modified this time frame to the past seven days in this RCT. K6 thus measured showed a Pearson correlations coefficient of 0.62 (95 % CI: 0.53 to 0.69, *p* < 0.001) with the standard self-rating depression instrument BDI-II and demonstrated similar sensitivity to change in this study [28].

### Treatment

The telephone CBT was a structured, manualised, eight-session program adapted from a previously established manual [34]. The participant and the therapist shared the patient manual containing all the materials for each session. A separate therapist manual was prepared specifying the procedure for each session. The participant and the therapist also shared a handbook that contained all the homework worksheets in a small notebook format.

Each session was designed to last 30 to 45 min, but the actual lengths varied according to the participants’ needs and the therapists’ assessment. Sessions were carried out at weekly intervals, but the scheduling was flexible. Each session began with an assessment of the participant’s distress symptoms during the past week using the K6 and a review of the previous session and the homework. The first session included psychoeducation regarding the CBT model and explained the theory of the whole program and gave the homework to self-monitor one’s own mood and associated thoughts. Sessions #2 through #4 focused on increasing pleasant activities through behavioral therapy (BT) [35]. Sessions #5 through #7 focused on cognitive therapy (CT) of negative automatic thoughts [36]. In Session #8 the participant and the therapist reviewed the behavioral and cognitive skills covered in the program and created a personal self-care plan. All the sessions included an assessment of enthusiasm in completing homework assignments in their daily lives, i.e. each participant was asked the two questions explained above for each homework.

The telephone therapists were clinical psychologists, social workers or nurses with at least 1 year of clinical experience. They were required to receive a didactic lecture, to listen to audiotaped sessions, and to treat two clients under supervision before they could participate as therapists. In addition, the quality of the administered tCBT sessions was assured by on-going supervision and consultation. Each participant received all the sessions from the same therapist. Because the original RCT compared the addition of tCBT to usual care against usual care alone, participants in both conditions were allowed to utilise the EAP provided by the company, which included stress diagnostics and a reduction program on the Internet, telephone consultation, and email consultation, when the employee desired. They were also free to seek any professional help, such as professional counselors and medical doctors. During tCBT, however, only one participant used telephone consultation once.

### Data analysis

We first summarised demographic and clinical characteristics of the participants at baseline and investigated if these variables (i.e., gender, age, job category, and job rank) correlated with variables of our interest, i.e. K6, Q1, or Q2 scores.

We conducted structural equation modeling (SEM) [37] to estimate whether and how the session-by-session enthusiasm to complete homework could predict subsequent session-by-session therapeutic outcomes. The session-by-session changes in K6 scores revealed, however, that most of the effect of the tCBT program was achieved by session #4: the change of mean K6 scores during the behavior therapy (sessions #2 through #4) was −1.75 points whereas that during the cognitive therapy (sessions #5 through #7 was −0.32 points only, which was less than one-fifth of that observed in the three sessions previously (Fig. 1). Thus the participants with subthreshold depression had already responded well to the initial behavior therapy interventions and there was not much variability left throughout the latter half of the program (floor effect). We therefore chose to examine enthusiasm ratings as predictors of symptoms for the behavior therapy sessions.

**Fig. 2:**
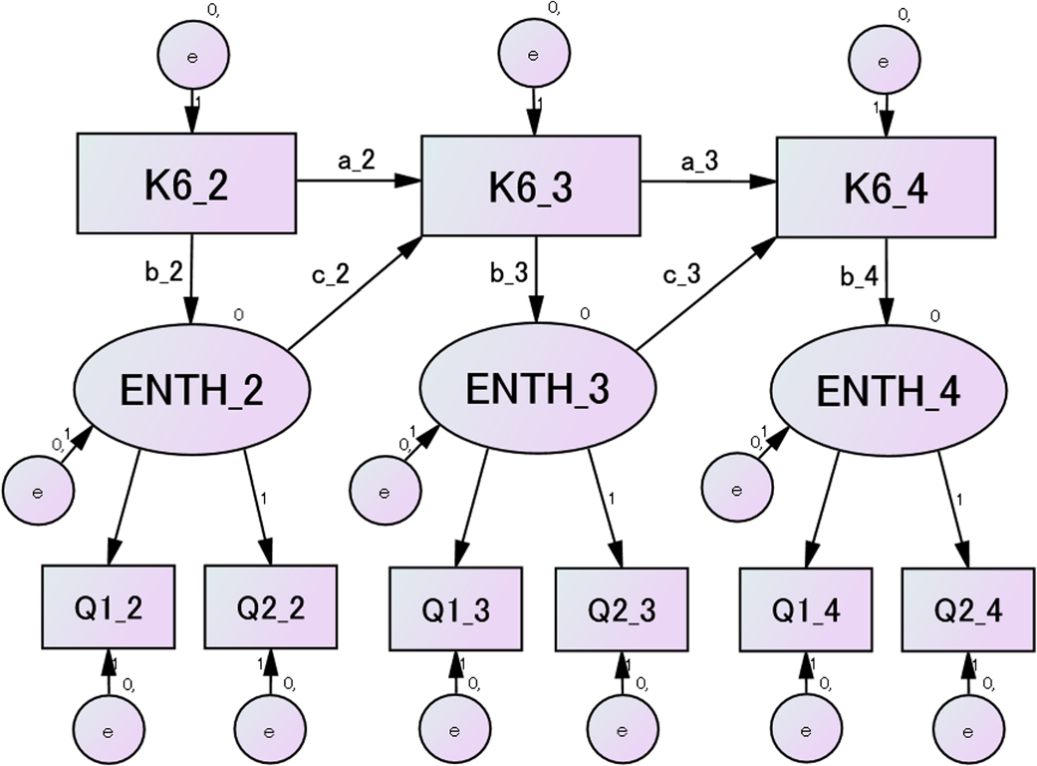
Model 1 hypothesized that the K6 score influenced enthusiasm during the same session, both of which then influenced the K6 score of the following session. K6_2 (_3) (_4): K6 score from 2nd (3rd) (4th) session. ENTH_2 (_3) (_4): Enthusiasm for homework of the 2nd (3rd) (4th) session. Q1_2 (_3) (_4): Q1 (see text) rating from the 2nd (3rd) (4th) session. Q2_2 (_3) (_4): Q2 rating from the 2nd (3rd) (4th) session. e: Error terms of the factors

**Fig. 3:**
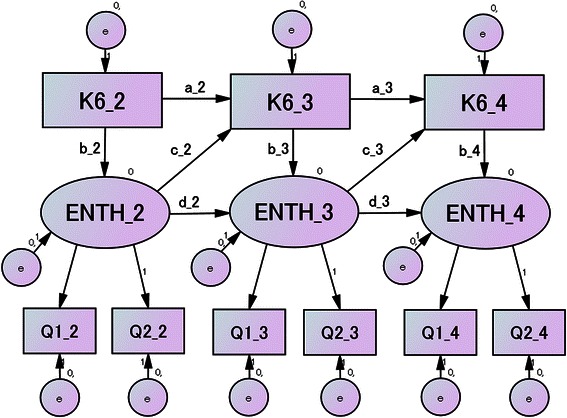
Model 2 further hypothesized that enthusiasm for the preceding session was correlated with enthusiasm for the following session

Figures 2 and 3 represent two SEM models that we examined in this study to explain the relationships among these variables. In both models, the responses to the two enthusiasm scores were supposed to be determined using an endogenous variable termed “enthusiasm” to complete the homework for each session. The enthusiasm and the K6 score of the previous session were then used to predict the K6 score of the subsequent session. We also hypothesized that enthusiasm could be influenced by the K6 score of the same session. In Model 2, we further hypothesized that enthusiasm at a prior session was correlated with subsequent enthusiasm.

For both models, we examined two further submodels with or without constraints, whereby coefficients for the corresponding correlations were held constant across sessions (It was hypothesized that paths a2 = a3, b2 = b3 = b4, c2 = c3, and d2 = d3 in the constrained model, but not in the unconstrained model).

The goodness-of-fit of the models was evaluated using the following indexes: chi-square test of the model (considered to be not a bad fit if *P* > 0.05), the Comparative Fit Index (CFI; considered to be a good fit if above 0.9), the Root Mean Square Error of Approximation Index (RMSEA; considered to be a good fit if below 0.05) and Tucker-Lewis Index (TLI; considered to be a good fit if above 0.95). We considered the RMSEA as the main index because it has been shown to be more sensitive than the others [38].

There is no standard method to calculate sample sizes required for SEM. SEM usually requires large samples, but required sample sizes depend on various characteristics of the model as well as what differences one wishes to detect in the analyses. Thus some suggest that SEM models can perform well even with relatively small samples (e.g., 50 to 100) while a larger sample (e.g., *n* > 200) would be desirable [39].

All statistical tests were two-sided, and p values less than 0.05 were considered statistically significant. We used SPSS and AMOS (Version 21.0; IBM Inc., Armonk, NY, USA) for all analyses.

## Results

### Patient characteristics

All in all 71 adults met the eligibility criteria for this study. Table 1 shows the demographic and clinical characteristics of the participants at baseline. The correlations between these baseline demographics (i.e., gender, age, job category, and job rank) and K6, Q1, Q2 scores at session 2 through 4 were in the ranges of 0.0 to 0.2 and were all non-significant.

**Table 1:** Demographic and clinical characteristics of the participants

Gender	Male	58 (81.7 %)
	Female	13 (18.3 %)
Age	Mean (Range, SD)	39.0 (23–57, 8.0)
Job category	Sales/Marketing	25 (35.2 %)
	Production/Factory	12 (16.9 %)
	Engineering/Technical	18 (25.4 %)
	Administration/Management	15 (21.1 %)
	Unkown	1 (1.4 %)
Job rank	Supervisory	18 (25.4 %)
	Nonsupervisory	53 (74.6 %)
K6 score	Mean at baseline (Range, SD)	9.8 (5–18, 3.4)
Enthusiasm	Mean of Q1 at baseline (Range, SD)	7.0 (1–10, 2.2)
	Mean of Q2 at baseline (Range, SD)	7.6 (0–10, 2.0)

Figure 1 showed change of the mean K6 score through the tCBT sessions. At baseline, the mean (range, SD) of the K6 score was 9.8 (5–18, 3.4), which dropped to 7.1 (0–22, 4.5) by session #4, with a pre-post effect size of 0.64. The mean (range, SD) of the first and the second questions to measure enthusiasm were 8.7 (4–10, 1.3) and 8.4 (5–10, 1.3) at session #2, 8.8 (6–10, 1.1) and 8.5 (5–10, 1.1) at session #3, and 8.6 (6–10, 1.3) and 8.1 (5–10, 1.3) at session #4, with Pearson correlation coefficients of 0.42, 0.46 and 0.57 between the first and second questions in the 2^nd^, 3^rd^ and 4^th^ session, respectively.

Five patients had dropped out by session #4. Five other patients had two missing values (Q1 and Q2 score for one of the sessions), and one patient had one missing value (Q2 score for session 4). Using the full information maximum likelihood method, we could include all participants in our analysis, and the final analysed sample size was 71.

### Comparison of different models

Table 2 shows the goodness-of-fit indexes of the four competing models, as described in the Materials and Methods section. An acceptable fit was obtained for both the unconstrained and constrained versions of Model 2, with the constrained model showing a slightly better fit than the unconstrained model. Consequently, we chose this hypothesized model as best representing the session-by-session relationships between enthusiasm and depressive symptom.

**Table 2:** Goodness-of-fit indexes for the four competing models

	*P* value for Chi-squared test	CFI^c^	RMSEA^d^	TLI^e^
Model 1, unconstrained	< 0.001	0.601	0.197	0.219
Model 1, constrained^a^	< 0.001	0.608	0.180	0.346
Model 2, unconstrained	0.366	0.990	0.033	0.978
Model 2, constrained^b^	0.400	0.992	0.025	0.987

^a^Hypothesized paths a, b and c were constant throughout the sessions

^b^Hypothesized paths a, b, c, and d were constant throughout the sessions

^c^Comparative Fit Index; considered to be a good fit if above 0.9

^d^Root Mean Square Error of Approximation Index; considered to be a good fit if below 0.05

^e^Tucker-Lewis Index; considered to be a good fit if above 0.95

**Fig. 4:**
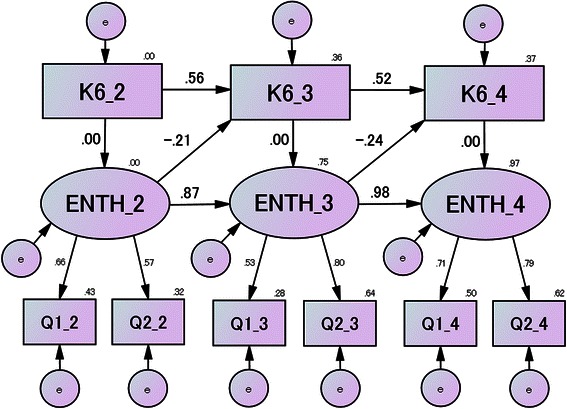
Standardized coefficients of the best-fitting model, Model 2, with constraints

Figure 4 shows the standardized coefficients for the final model. The correlation coefficients of the three paths were statistically significant, but path b was not (*P* < 0.001 for path a, *P* = 0.009 for path c, and *P* < 0.001 for path d, but *P* = 0.96 for path b). Thus, the K6 score measured at the beginning of the session did not influence the enthusiasm to complete the homework for that session. Both the K6 and the enthusiasm scores of the preceding session strongly and positively predicted the respective K6 and enthusiasm scores for the subsequent session, while enthusiasm to complete homework negatively predicted, i.e., decreased, the K6 score of the subsequent session.

## Discussion

The present study is the first to examine the session-by-session relationships between enthusiasm to engage in homework and changes in psychological distress through the course of behavior therapy for subthreshold depression using structural equation modeling. The best fitting model suggested that distress severity at the beginning of a session did not influence the enthusiasm for completing the homework from that session (estimated r = 0.0), which then, however, was negatively associated with the distress severity of the following session (r = −0.21 to −0.24).

The largest strength of this research is the examination of the session-by-session relationships between the enthusiasm to complete homework and psychological distress severity. A number of preceding studies have shown that homework in CBT was associated with the improvement of depression and anxiety; in these studies, however, homework compliance was measured either as average throughout the CBT sessions or at one out of the many sessions, while depression was typically measured at the end of treatment. In this study, by contrast, we examined the session-by-session relationships between homework and distress and found that enthusiasm to complete homework did indeed lead to a reduction in distress at the time of the subsequent session.

Another strength of our research is that we measured enthusiasm to engage in homework at the end of each session with regard to the particular homework assignments of that session. During the sessions, the therapists used Socratic questions to enhance the clients’ motivations while also modifying the assignments and troubleshooting possible barriers if necessary. In other words, in contrast with prior studies, which studied homework compliance in terms of the completion of the assigned homework ex post facto, we measured a variable that therapists can work on collaboratively with their clients and found that such enthusiasm did lead to distress symptoms reduction during the following week. It is also important to note that this enthusiasm was independent of the distress measured at the beginning of the session. In other words, therapists can strive to enhance the clients’ enthusiasm for homework regardless of the initial psychological distress severity.

It is important to note that not only distress, but also enthusiasm thus measured showed strong session-to-session correlations. It is natural to expect that distress severity at one session would predict distress severity at the subsequent session. The very strong correlation between enthusiasm measurements throughout the sessions may be a reflection of the patient’s personality (e.g., tenacity to engage in assignments), his/her determination and preference for BT, and/or a stable collaborative relationship between the therapist and client. One could argue that there may be little room for the clinician to work on, if the former were the only factor behind the observed strong correlations. The observational nature of the current study precludes any further elucidation in this regard. However, our study does suggest that enhancing this factor through a collaborative therapist-patient relationship could contribute to a reduction in distress in the coming week.

There are several limitations in this study that should be acknowledged. First, as this research was a secondary analysis of a completed trial, the sample size was limited by the available data and was not as large as one would have preferred. This may have led to some possible type II errors (dismissing true associations). On the other hand, we were able to observe several statistically significant correlations that would be clinically meaningful. Second, as our sample consisted of employees with subthreshold depression who were not seeking clinical help and who were working, our results may not be readily generalizable to moderately or severely depressed clinical cases. Moreover, the majority of our samples were men, and this would also affect to generalizability. This limitation was primarily due to the high proportion of male employees in the company where the original RCT took place. Third, as the participants had made most of their improvement through the BT sessions alone, our session-by-session analyses of the relationships between homework enthusiasm and improvement in psychological distress were limited to the BT sessions and we could not investigate them through the CT sessions conducted in the latter half of the program. Thus our results would apply to BT homeworks but might not to CBT homeworks in general. Forth, although we estimated enthusiasm using two questions with satisfactory internal consistency reliability, as described in the Methods section, there is no standardised method to measure enthusiasm, so more refinement in this direction is desirable. Fifth, client characteristics other than enthusiasm and level of distress, such as perfectionism and fear of failure [20], and therapist characteristics were not examined in the present study. Further research is needed to examine interplay of such characteristics for homework completion and improvement of psychological distress or depression.

## Conclusion

In conclusion, the clinical and research implications of the present study may be as follows. Clinicians now have empirical evidence when they strive to work collaboratively with their clients to enhance their enthusiasm for homework, since it was shown that a higher enthusiasm leads to a reduction in distress during the subsequent week; moreover, they can and should do this regardless of the clients’ distress severity at that session because these two factors are not correlated. Research into homework in CBT should no longer be content in making global measurements once at the end of the treatment, but such research should examine its vicissitudes and mutual influences throughout the sessions using statistical methods that allow longitudinal modeling. Only then can such research illuminate what each clinician and patient can do in each session.

## Appendix: Figure 1

Participants flow diagram.

## Abbreviations

BDI2: Beck depression inventory-II
CFI: Comparative fit index
CIDI: Composite International Diagnostic Instrument
EAP: Employee assistance program
RMSEA: Root mean square error of approximation index
SEM: Structural equation modeling
tCBT: Cognitive behavioral therapy via telephone
